# Health Beliefs and Practices Related to Dengue Fever: A Focus Group Study

**DOI:** 10.1371/journal.pntd.0002310

**Published:** 2013-07-11

**Authors:** Li Ping Wong, Sazaly AbuBakar

**Affiliations:** 1 Department of Social and Preventive Medicine, Faculty of Medicine, University of Malaya, Kuala Lumpur, Malaysia; 2 Julius Centre University of Malaya (JCUM), University of Malaya, Kuala Lumpur, Malaysia; 3 Department of Medical Microbiology, Faculty of Medicine, University of Malaya, Kuala Lumpur, Malaysia; 4 Tropical Infectious Diseases Research and Educational Centre (TIDREC), University of Malaya, Kuala Lumpur, Malaysia; Pediatric Dengue Vaccine Initiative, United States of America

## Abstract

**Background:**

This qualitative study aimed to provide an in-depth understanding of the meaning of dengue fever (DF) amongst people living in a dengue endemic region, dengue prevention and treatment-seeking behaviours. The Health Belief Model was used as a framework to explore and understand dengue prevention behaviours.

**Methods:**

A total of 14 focus group discussions were conducted with 84 Malaysian citizens of different socio-demographic backgrounds between 16^th^ December, 2011 and 12^th^ May, 2012.

**Results:**

The study revealed that awareness about DF and prevention measures were high. The pathophysiology of dengue especially dengue haemorrhagic fever (DHF) and dengue shock syndrome (DSS) were rarely known; as a result, it was seen as deadly by some but was also perceived as easily curable by others without a basis of understanding. Young adults and elderly participants had a low perception of susceptibility to DF. In general, the low perceived susceptibility emerged as two themes, namely a perceived natural ability to withstand infection and a low risk of being in contact with the dengue virus vector, *Aedes spp.* mosquitoes. The barriers to sustained self-prevention against dengue prevention that emerged in focus groups were: i) lack of self-efficacy, ii) lack of perceived benefit, iii) low perceived susceptibility, and iv) unsure perceived susceptibility. Low perceived benefit of continued dengue prevention practices was a result of lack of concerted action against dengue in their neighborhood. Traditional medical practices and home remedies were widely perceived and experienced as efficacious in treating DF.

**Conclusion:**

Behavioural change towards attaining sustainability in dengue preventive practices may be enhanced by fostering comprehensive knowledge of dengue and a change in health beliefs. Wide use of unconventional therapy for DF warrants the need to enlighten the public to limit their reliance on unproven alternative treatments.

## Author Summary

In-depth understanding of health beliefs and behaviors may provide insights into sustainable community-based dengue prevention and control. This study uses qualitative method to explore dengue prevention and treatment-seeking behaviours. Focus group discussions were conducted with Malaysian public of various demographic backgrounds in Klang Valley, Malaysia. General knowledge about dengue fever was good but many lack of knowledge of dengue haemorrhagic fever (DHF) and dengue shock syndrome (DSS). Many had low perceived susceptibility of getting dengue. Barriers to prevent dengue were categorised as low self efficacy to execute preventive measures, perceived lack benefit of individual preventive measures, and unsure susceptibility of getting dengue. Low perceived benefit of continued dengue prevention practices was a result of lack of concerted action against dengue in their neighborhood. Traditional medicinal practices, though unproven, were common and viewed as efficacious. The findings add valuable insights into how health beliefs could affect dengue prevention and control in endemic region.

## Introduction

Since the beginning of the 21st century, dengue fever (DF) have been the most important vector-borne arboviral disease in humans, occurring mainly in tropical and sub-tropical countries where over 2.5 billion people are at risk of infection [1]. With an estimated 50–100 million dengue infections worldwide, the disease is currently endemic in more than 125 countries in Africa, the Americas, the Eastern Mediterranean, South-east Asia, and the Western Pacific [1]. The incidence of DF in Malaysia has increased dramatically in recent decades and has remained a serious public health problem. Since the first reported outbreak in 1902, several major outbreaks were reported including in 1974, 1978, 1982, and 1990 [2]. The dengue incidence rate increased from 44.3 cases/100,000 population in 1999 to 181 cases/100,000 population in 2007, which exceeded the national target for the incidence rate of DF and dengue haemorrhagic fever DHF of less than 50 cases/100,000 population. An increase in dengue deaths in the adult population has been observed since 2002. The case fatality rates, however, for both DF and DHF remained below 0.3% [3]. The highest age-specific morbidity rates were in the 15 to 35 years age groups [4].

Preventing or reducing dengue virus transmission depends largely on controlling the mosquito vectors. As such, in most dengue endemic countries, anti-dengue campaign involved media bombardment with messages to eradicate the *Aedes spp.* mosquitoes to the point that *Aedes spp.* is synonymous with dengue. Human behaviours and activities, as well as demographic, social and possibly climate changes also contribute greatly to the increased incidence and geographical spread of the disease. This has led to the introduction of Communication for Behavioral Impact (COMBI). Although community-wide effort is the key to eradicate dengue, commitment and participation at individual level such as emptying flower pots and practice of regular removing of water collecting containers and rubbish from their homes, play equally critical role. The Health Belief Model (HBM) is one of the most widely used social cognition models to predict health behaviours. The HBM posits that individual's health behaviour is determined by four main elements: i) consideration of the likelihood (susceptibility); ii) consideration of the seriousness (severity) of illness; iii) perceived benefits of taking health action; iv) perceived barriers to taking health action. These four perceptions are elements that determine the readiness to take action, and are activated by: i) cues to action and ii) self efficacy [5]–[6]. There have been a handful of studies that have specifically applied the HBM in attempts to understand perceptions of risk and sustained dengue prevention [7]–[9]. HBM has also been used as a framework for understanding how to effectively structure health communication messages in order to change individual behaviour to prevent dengue [10].

To date, exploration of the constructs of HBM in the context of dengue prevention in the majority of research literature is limited to the realm of quantitative research. Only a few qualitative studies have examined health perceptions of DF [7], [11]–[12]. These studies underline the importance of public health beliefs in determining preventive measures against DF. However, these studies have been limited by lack of in-depth explorations of the main four dimensions of the HBM and its specific link to dengue prevention practices.

Additionally, within the study of health behaviours, knowledge about dengue and its association with preventive behaviours have not been adequately elucidated. Findings have shown that increased understanding of dengue virus transmission was positively attributed to better dengue prevention practices [13]–[14]. Nevertheless, there were evidences which imply that knowledge about DF does not always result in the adoption of recommended preventive behaviours [15]–[16] and thus, further in-depth understanding among those living in dengue endemic country is warranted.

Due to its relative scarcity in the research literature, we undertook this qualitative study as a first step in gaining in-depth understanding of the meaning of dengue, dengue prevention and treatment-seeking behaviours in a dengue endemic country. The HBM was used as a framework to explore and understand dengue preventive health behaviours. Qualitative exploration of attitudes and beliefs using constructs from the HBM has the potential to develop an in-depth understanding of the complex interplay of psychological, social, cultural and individuals factors that give rise to differences in dengue related health behaviours.

## Methods

### Ethics Statement

Participation in this study was voluntary and all participants provided written informed consent. All information was collected anonymously and the outcomes were used for research purposes only. The study was approved by the Medical Ethics Committee of the University of Malaya Medical Centre, Kuala Lumpur, Malaysia (MEC Ref No. 896.15).

### Study Sample

Participants were members of the public in Malaysia. A sample of multi-ethnic Malaysian citizens with diverse educational and socio-economic backgrounds were recruited in the Klang Valley area of Malaysia, the locale with the highest annual incidence of dengue. The target participants were selected based on the convenience sampling method at the research area. The first step in the recruitment of focus group participants began with word of mouth referral and through personal contacts by research assistants. Once recruited, participants were screened for eligibility using the following inclusion criteria: i) aged 18 years or over, ii) willing and able to provide written informed consent, iii) living in the Klang Valley and iv) Malaysian citizen. Subsequently, the focus group participants were asked to refer us to other participants they knew who also met the inclusion criteria, such as their friends or acquaintances, via the snowball sampling method.

### Data Collection

A semi-structured focus group moderator's guide corresponding to the research questions was developed. This semi-structured guide allowed the moderator to pose questions that flowed from one issue to the next. The guide consisted of questions about i) the meaning of DF and general knowledge about dengue prevention and treatment, 2) attitudes about dengue with probes on perceived severity and susceptibility of dengue, 3) prevention practices and barriers to prevention, and 4) treatment-seeking behaviours. Discussion probes based on HBM constructs were developed to facilitate discussion on barriers to prevention. Focus group discussions (FGDs) were conducted in community settings and at places that were convenient for the participants, such as their home or workplace. Groups were separated into the three main ethnic groups of Malaysia, Malay, Chinese and Indian and were conducted in the local languages of the participants. Besides ethnic diversity, participants representing a broad array of socio-economic backgrounds within each ethnic group were recruited to allow exploration of differences in groups from different socio-economic backgrounds.

Written informed consent was obtained from all participants prior to the FGDs. All discussions lasted approximately 45 minutes, and were audio-taped and transcribed verbatim. FGDs conducted in languages other than English were forward translated into English. Notes taken by the moderator and note taker supplemented the audio-taped transcripts to glean details from the discussion. After group discussion, a brief questionnaire was administered to participants to gather information regarding their demographic backgrounds.

### Data Analyses

The sampling process, data collection and analyses were continuous and iterative. All group discussions were immediately analysed and compared with the analysis of the previous discussions, which, in turn, further shaped the subsequent sampling, data collection and analysis. The FGDs continued until data saturation was reached or no new information was uncovered. After transcription and cleansing, the transcripts were converted to rich text format and imported into NVivo software (QSR International Pty Ltd, Doncaster, Victoria, Australia) for coding and categorising [17].

A directed content analysis approach was used to analyse the data and to identify key themes relating to the HBM constructs, knowledge and treatment-seeking behaviours [18]. The data were first segregated using predefined initial codes corresponding to the determinants of HBM. Subsequently open coding was employed to identify themes emerged under the concept, and more specific axial codes were thereafter developed from the open codes [19]. The codes were analysed using an interpretive descriptive method, where interpretative description goes beyond a mere description and aims to provide an in-depth conceptual understanding of a phenomenon [20]. Coding was performed by a single coder and the consistency of coding was assessed by intra-coder reliability. The researcher coded segments of the data at two different periods and the intra-coder reliability was calculated as the number of agreements divided by the total number of agreements and disagreements. The calculated intra-rater agreement was in the 90th percentile range. Finally, the data were interpreted and presented using the participants' own words as illustrations.

## Results

### Participants

A total of 14 FGDs comprising 5 Malay, 5 Chinese and 4 Indian groups were conducted between 16^th^ December, 2011 and 12^th^ May, 2012. Each FGD was composed of between 5 and 8 participants of the same ethnic group (total 84 participants). The mean (± SD) age of the sample was 39.8 (±15.8) years, age range 21 to 70 years old. The demographic distribution of the study sample is shown in Table 1. Most of the study participants had at least a high school education. The FGDs comprised housewives, students, unemployed and employed persons of various occupational categories in managerial, professional and technical-unskilled workers. Among all participants in the FGDs, 7 persons reported that they had dengue experience. The average time for one FGD was approximately one hour. All the groups contributed valuable and important information on all the issues being discussed during the discussions. Table 2 summarizes the major themes derived from the FGDs.

**Table 1:** Demographic characteristic of the participants.

*Personal background*	Total n(%)	Focus group (n)													
		1	2	3	4	5	6	7	8	9	10	11	12	13	14
Age															
30 and below	33(39.3)	7	4			6		2	8						6
31–60	35(41.7)		1		7		8	4		5	5		5		
Above 60	16(19.0)			5								5		6	
Gender															
Male	38(45.2)	7					8	6	8		5	5			6
Female	46(54.8)		5	5	7	6				5			5	6	
Educational level															
Primary	6(7.1)				1							5			
Secondary	32(38.1)			4	5		6	3		5	2		3	4	
Tertiary	46(54.8)	7	5	1	1	6	2	3	8		3		2	2	6
Ethnicity															
Malay	33(39.3)	7			7		8	6				5			
Chinese	28(33.3)					6					5		5	6	6
Indian	23(27.4)		5	5					8	5					
***Family background***															
Average household monthly income^a^															
RM1000 and below	16(19)	2		4			1		2	1		4		2	
RM1001-3000	41(48.8)	3	4	1	6	6	6	3	4	2		1			5
Above RM3000	27(32.1)	2	1		1		1	3	2	2	5		5	4	1
Locality															
Urban	62(73.8)	4	4	5	5		7	4	6	4	3	5	4	5	
Suburban	12(14.3)	2	1		1		1	2		1	2		1	1	
Rural	10(11.9)	1			1	6			2						6
***Experience***															
Dengue fever experience															
Yes	7(8.3)		3	1						1				1	1
No	77(91.7)														
Frequency dengue exposure															
Once	5(6)		1	1						1				1	1
Twice	2(2.4)		2												
Hospitalized because of dengue															
Yes	6(7.1)		3	1										1	1
No	78(92.9)														

a 1 US Dollar = 3.0 Malaysian Ringgit (MYR).

**Table 2:** Summary of key findings of the main themes.

Theme area probed in discussion	Key themes identified
**Knowledge and Awareness**	Knowledge about sign and prevention. Knowledge prolonged fever is a common sign and remove sources of stagnant water to prevent mosquito breeding.
	Knowledge about severity. Know dengue is deadly but little knowledge about dengue pathogenesis.
	Knowledge of traditional medicine practices.
**Attitudes towards dengue**	
***Perceived Severity***	Serious or highly deadly.
	Not a threat. Perceived unsusceptible.
***Perceived Susceptibility***	Susceptible.
	Perceived little or no chance. Perceived not at risk.
**Prevention Practices**	Prevent against mosquito bite.
	Destroy mosquito breeding site.
**Barriers to prevention**	
***Self efficacy***	Lazy or forgotten to practice prevention
***Perceived benefit***	Lack of community converted effort, therefore individual effort not beneficial
***Perceived severity***	No report of dengue cases imply less severity
***Perceived susceptible***	Perceived low susceptibility associated to perceived immune to infection.
	Unsure of perceived susceptibility as dengue could happen by chance or luck.
**Treatment seeking**	Modern medical treatment.
	Traditional treatment (natural remedies, traditional medicine). Reason for using traditional and natural remedies; i) perceived helpfulness, ii) trust of natural treatment and iii) pragmatic to use.
	Dengue cases in neighborhood, and community as cue to action to seek immediate medical attention.

### Knowledge and Awareness about Dengue

Some participants described DF as “dangerous” but were not clear about how dengue can lead to death. The vast majority of participants knew that DF fever is caused by mosquitoes. Participants above the age of 60 years with low levels of education had limited knowledge that DF is specifically caused by a mosquito infected with the dengue virus. Many were unable to differentiate between DF and DHF, including some of the highly educated participants. Those who knew about DHF described DHF as “difficult to be cured”. The most salient theme emerging across the focus groups was the notion that the dengue virus will not spread to a person with a strong immune system when bitten by an infected mosquito. Alternatively, they viewed people with a weak immune system will not be able to resist dengue infection after being bitten by a dengue mosquito.

“*My roommate, a few of my friends and many others got it (DF), many mosquitoes here, but I didn't get. I am healthy, my body is strong, because I exercise frequently.*” Malay male, 23 years old.

The focus group participants were easily able to identify the breeding source of mosquitoes. Almost all knew that dengue mosquitoes breed in stagnant water and that they should frequently check and remove stagnant water to prevent mosquito breeding. The majority noted that the signs and symptoms of dengue was a raised body temperature or prolonged fever. Some knew and cited that DF can be confused with other febrile respiratory illnesses as they share some of the same symptoms. Participants with a higher education were more likely to be able to correctly identify other symptoms such as rashes, joint pains and muscle pain. Dengue-experienced participants had relatively greater knowledge of the signs and symptoms than those who never had dengue. Overall, there were no differences between groups of different ethnic composition in knowledge about dengue and dengue prevention.

When the study participants were asked to share their knowledge regarding treatment for dengue, there appeared to be a tendency for participants of various ethnic and demographic levels across all discussion groups to mention the efficacy of 100Plus (a brand of carbonated isotonic sports drink) in treating DF. Although participants in general did not know the mechanisms of action of 100Plus in treating DF, many appeared to have heard of its efficacy through word of mouth. Other commonly mentioned treatments for DF were extracts or juices of vegetables or fruits. . Across the focus groups, a considerable number of participants reported that they had heard of extract from papaya leaves as effective for curing DF. Many also cited that they had heard of watermelon juice as another cure for DF. Several other participants mentioned that bitter gourd juice has also been used to treat DF.

Frog soup was commonly cited by Chinese participants, whereas the Indian and Malay participants were more likely to have heard that crab soup helps to relief symptoms of DF. A considerable number of Chinese participants strongly believed that porcupine bezoar stone is effective in treating DF.

“*Hao Zhu San* (porcupine bezoar stone) *is effective, it is very expensive. My friend had dengue and took it, just a bit, put under the tongue, then chew and swallow, and drink a lot of water. Like that a little bit* (showing the tip of a finger) *already MYR200 to MYR300. Can easily find in Chinese medicine shops, but have to beware of the fake one nowadays.*” Chinese male, 41 years old.

Some of the focus group participants implied that the natural remedies that they cited have been commonly used as treatment for dengue instead of as a relief of DF symptoms. Some of the responses implied that patients have been cured of dengue by these natural remedies. As one woman said,

“*Crab soup. Boil the whole crab, and the shell, not just meat. From what I see, many people who have dengue that take crab soup recover after taking.*” Malay female, 24 years old.

It was also revealed that among the participants who knew or had heard of the efficacy of relieving DF symptoms using natural remedies, or traditional or unconventional treatments, they tended to have preconceived ideas that dengue is not as dangerous as other diseases that warrant professional medical treatments.

### Attitudes: Perceived Severity and Susceptibility of Dengue

The perception of severity of dengue described by the participants fell into two main themes: serious or highly deadly, and not a threat. Participants who described dengue as serious and highly deadly comprised approximately one-third of the total number of FGD participants, most of whom knew of neighbours, friends or counterparts who had died from dengue. They viewed dengue as a fast killer illness and the person that they knew or had heard about who had contracted dengue died within a few days after being admitted to the hospital. The remaining FGD participants who viewed dengue as not a threat were relatively younger, mainly between the ages of 18 and 35 years old. They viewed that dengue is not dangerous if a person seeks treatment early and perceived that death only occurs among those who do not seek proper medical care.

“*If you go early to see doctor, then should be alright, just few days fever.*” Malay male, 23 years old.

The focus group participants reported a mixed view when queried on their perceived susceptibility of contracting dengue. They viewed dengue as very common in Malaysia and dengue infection as widespread in many areas. A minority of the focus group participants perceived little or no chance that they would contract dengue. They believed that dengue occurs because of bad luck, chance, fate or uncontrollable factors. They reasoned that dengue is unlike other contagious or infectious diseases where a person is at a particularly high risk of becoming infected upon contact with an infectious agent on the body surface. Only the Aedes mosquito and not all mosquito species can transmit dengue, and even if a person is bitten by an Aedes mosquito, only dengue virus infected mosquitos will cause dengue. Some participants stated that their risk of getting dengue was low as they were confident that the mosquitoes in their area were not Aedes mosquitoes.

*“The mosquitoes here are from the jungle, they are not dangerous. Every evening they come, we are used to it, mosquito bites are common, we turn on the fan every night.”* Malay female, 50 years old, housewife.

Furthermore, some viewed that if a person has a strong body defense, a dengue virus infected mosquito will not cause DF in the person. There were also several participants, a majority of whom were young male, who were confident that they have strong body defenses against dengue and will not contract dengue. They viewed that many people who contracted dengue were because their body defenses were not as strong.

“*The place I stay…very likely….because there is a lake, water stagnant. Many cases, I may get dengue, but I think I am at low risk, because my body defense is strong and I am healthy.*” Indian male, 21 years old, college student.

“*Alhamdulillah (Thank god), I have never had dengue, all around me in the kampong* (village), *my own brother had, but I kebal* (tough).” Malay male, 32 years old.

An elderly participant noted that mosquitoes prefer young children over elderly people. And that the hardened skin layer of an elderly serve as barrier against mosquito bites.

*“The mosquitoes prefer to bite small children than old people. Our skin thick and hard, cannot go through.”* Malay male, 69 years old.

### Dengue Prevention Practices and Barriers

When asked about measures to avoid mosquito bites, personal protective measures mentioned by the participants were the use of insecticide sprays, electric rackets, mosquito coils, electric vaporising mats, a mosquito net around bed or installed in windows, protective clothing (wearing a long-sleeved shirt and long trousers when they go to mosquito-infested areas), and avoiding being outdoors at dusk and early evening. Concerns about undesirable hazards relating to mosquito coil smoke or electric mat vapour inhalation were expressed across the focus groups and posed barriers to their consistent use. Many participants told of their preference for non-chemical control alternatives or natural methods to repel mosquitoes, as implied by the following quotations:

*“I drank ubat kampong* (folk medicine) *my body becomes bitter….blood becomes bitter. The mosquitos won't bite. That herb… we plant in the village, very bitter, the name is Bakawali (Tinospora spp.). We mixed it with pucuk mengkudu* (*Morinda spp.* shoots), *daun asam belimbing* (Averrhoa bilimbi leaves), *sireh* (Piper betel). *Last time, I used to go in and out of the forest, the Orang Asli* (aborigines) *taught us about this.*” Malay male, 69 years old, retired.

“*I apply lemon grass oil, the mosquitos do not like, it can prevent me from mosquito bite*” Malay female, 50 years old, housewife.

“*I bought a plant from an agricultural exhibition, it can help keep mosquitoes away. I put it outside the window.*” Malay female, 50 years old, housewife.

Various means of destroying mosquito breeding were also cited, such as frequent checking and removing stagnant water from containers in their homes and covering water containers to reduce mosquito breeding. When the focus group participants were probed using a series of questions regarding their barriers to dengue preventive practices, four themes emerged during coding. The barriers to sustained dengue prevention that emerged across all the focus groups of different ethnic composition, in the order of the most commonly appear themes, were; i) lack of self-efficacy, ii) lack of perceived benefit, iii) low perceived susceptibility, and iv) unsure perceived susceptibility. A perceived lack of self-efficacy emerged in almost all focus groups and across all ethnic and demographic characteristics. The participants admitted to the challenge of constantly keeping the environment clean and free of mosquito breeding sites. Findings from the focus groups indicated that most participants, regardless of ethnicity, failed to constantly change stagnant water in pots and vases and check for mosquito breeding sites. Participants reasoned that they either forgotten or lazy to practice. Many revealed that if dengue cases are reported, the community gears up and cleans the surrounding environment. Taken together this implies lack of self efficacy and low perceived severity of outbreak influence prevention practices.

“*If nothing happens, we just relax (take it easy). Usually after a couple of months later, everybody relax and go back to square one*” Chinese female, 60 years old, housewife.

The lack of perceived benefit manifested itself as a perceived lack of control of their chance of getting dengue. The focus group participants viewed that prevention practices may not minimise their chances of getting dengue. They reasoned that the root cause for the spread of dengue is beyond their control. A participant whose home is a mosquito-affected area due to its location next to a forest said,

“*My house has a lot of mosquitoes, because the other side is jungle, we cannot do anything. Everyday we are troubled by mosquitoes. We can only close the doors and windows to prevent mosquitoes from coming in*” Chinese female, 35 years old, housewife.

Some stated that despite good self-practices to prevent dengue infection around their homes, such behaviour does not yield benefit as there is lack of a concerted effort from neighbours or other community members. As two participants stated,

“*It is not from our house, I think it is from somewhere else. I think because of a bistro is there, you know the restaurants. They didn't have proper drainage, so all things are blocked there. So, even if we clean our house, other places breed the mosquitoes,… no point.*” Indian female, 64 years old, housewife.

Low perceived susceptibility manifested itself as a perceived lack of changes of getting dengue even if they are in a high risk area or exposed to mosquito bites. A participant responded, when asked about the perceived susceptibility of getting dengue:

“*I am not at risk! Because I am an athlete and my body immune system is strong. It is more dangerous to the elderly and children.*” Indian male, 21 years old, student.

Uncertainty about how susceptible a person is to contracting dengue has also emerged as a theme, such that some participants felt that they were not in total control in avoiding dengue. One such participant explained that despite a good dengue preventive practice at home, it is still possible to contract dengue elsewhere in other areas, and therefore practicing preventive measures may not minimise the chances of contracting dengue.

“*It depends on takdir* (fate), *you cannot escape a destiny if the are fated to get dengue. My sister got it from her school.*” Malay female, 24 years old, university student.

Another participant added,

“*Even I am staying in Puchong, the mosquito travel from Kelana Jaya* (16 kilometers apart) *to bite me. So I have the potential to get the infection*”. Indian female, 27 years old.

### Treatment-Seeking

Dengue cases reported in the community appeared as cue to action in seeking immediate medical attention if they fall sick, as illustrated in the following quote

“*If fever, take panadol only, unless if we hear people have dengue fever nearby us, we sure afraid and suspect, in that case we immediately go to check for dengue.*” Chinese male, 45 years old

Most participants stated spontaneously that people who are ill with symptoms of suspected dengue infection should seek modern medical treatment. All the participants who had dengue experience reported that they sought modern medical treatments. While the majority emphasized the importance of modern medical treatment, several participants noted that, to some extent, it was necessary to simultaneously try various alternative treatments as they knew there are no specific treatments for dengue. Many related their experiences of how natural remedies do indeed have a healing effect against dengue. As one participant stated,

“*The frog soup, I heard from my friends, they had dengue, so they told us what to do, we just try and it really helps.*” Chinese female, 69 years old.

When participants who had never experienced dengue were asked for their perceived treatment-seeking practices for dengue, many of the participants' narratives suggested that there was an interest and belief in effective means for seeking modern medical care. Although the majority favoured modern over traditional practices or folk medicines in treating dengue, a considerable number of participants noted that they were likely to try various alternative treatments if they were suspected to have dengue. Across the focus groups, most of the participants who had never had dengue unanimously opined that folk medicines or natural remedies should be used in addition to conventional Western practices. The participants' reasons for using folk medicines and natural remedies for dengue can be classified into three main categories, ordered from most to least frequently occurring themes: i) perceived helpfulness, ii) trust of natural treatment and iii) pragmatic to use.

The narratives from the focus group participants suggested that high perceived helpfulness or efficacy of alternative treatment was related to word of mouth from local communities that had experience DF. Despite the lack of scientific evidence to support the efficacious claims of these natural remedies, also referred to as folk medicines, many participants reported that they have heard from others that the treatments effectively heal DF and therefore, they have a strong belief in the effectiveness of these treatments. These themes particularly emerged from focus group participants of lower educational level.

“*I have heard, that time the person has dengue fever, but he did not need to see a doctor, the fever continues. He took crab soup and subsequently he recovered!*” Malay male, 63 years old.

“*This healing crab soup, actually was told to us by the elderly folks. So, we try and we give to whom that down with dengue.*” Malay female, 37 years old.

The second theme, trust of natural treatment, derived from the notion that natural treatments have no potential harmful side effects. Many of these are home remedies that are easily available and prepared at home. One participant stated,

“*All these natural treatment are normal household items. For example, 100Plus has salt, dengue patients usually their antibody is not strong, so the salt will help to increase energy. Even doctor also advise to take.*” Malay male, 59 years old.

Thirdly, the pragmatic use of natural and traditional therapies, a theme that emerged in the focus groups with higher educated participants, largely derived from the notion of knowing that there is no specific cure for the dengue. One participant stated that it is sensible to turn to complementary medicines and natural remedies as only intravenous drips were given if a person is admitted to hospital. He said,

*“Nothing much can be done. If admitted in the hospital, will only be put on drips to provide them with fluids, don't want to get dehydrated. Since there is no other way, why not* (practice alternative remedies)?” Chinese male, 45 years old.

## Discussion

In our study, we found that most of the study participants have good knowledge about the signs and symptoms of dengue infection, transmission, and dengue prevention and control. However, many have little knowledge about DHF/DSS and are unable to differentiate it from DF. Although many viewed DF as a potentially fatal disease, to most participants, DF can often be easily cured by receiving an intravenous fluid therapy in the hospital. Many held onto their opinion that death due to dengue is related to a delay in treatment. The study also found a common belief that the deadly connotation of dengue was often the result of one's poor body immune system. As a result, healthy young male participants were more likely to have a low perceived vulnerability to DF. The view that being young and having a strong immune system will protect against dengue infection was a common testimonial among young male participants. And this is disconcerting considering that dengue incidence is the highest among the 15 to 35 years old. There were no differences in perception of susceptibility and belief among the young of the different ethnic background. Though several genetic studies [21], [22], attempting to correlate susceptibility to severe dengue and HLA types have identified genetic variants associated with increased susceptibility to dengue. There is however, a considerable debate surrounding the issue of genetic variation among humans in their susceptibility to dengue virus infection [23]–[25]. Our findings suggesting the nonchalant attitude toward dengue among the young could reshape our approach on message delivery to this vulnerable target population.

The good knowledge about dengue prevention and control among the study participants could be the result of mass media anti-dengue campaign. The campaign message usually focuses on community mobilisation activities to eradicate mosquito breeding. This is likely considering Klang Valley, the current study site, has the highest annual incidence of dengue and therefore, is more likely to have seen dengue containment campaign and activities. Nevertheless, a message about dengue pathogenesis which was lacking in the anti-dengue campaign could have resulted in the lack of knowledge among most respondents regarding DHF and the risk of contracting DHF following secondary dengue infection. Previous findings have indicated that being knowledgeable about dengue signs and symptoms, management and transmission is not associated with taking precautions against dengue [15]–[16]. Taken together, this evidence may imply that awareness campaigns should also impart knowledge of dengue pathophysiology, with particular emphasis on the vulnerability of a person to develop DH regardless of age and its severe manifestations such as DHF especially in secondary dengue.

It is interesting and important to note that this study also uncovered the narrative views from elderly participants who believed that thickened and hardened skin of elderly person is a natural barrier against mosquito bites and that elderly people are therefore, less likely to be bitten, and they underestimated the risk of dengue infection. Adults and elderly participants viewed children as particularly vulnerable to dengue infections. Such misperceptions resulted in a sense of fearlessness to DF and the perception of a lack of susceptibility to dengue infection, especially among young adults and the elderly, leading to a lack of cautionary measures. Such misguided beliefs are important key factors to be taken into account when planning tailored interventions, particularly for adult males and the elderly.

Mosquitoes and other rain forest insects are common in Malaysia as about 60% of Malaysia consists of tropical rain forest. One unanticipated finding was that some of the participants from rural areas viewed mosquito menace as common in their daily life and believed that nothing can be done about it. Participants who lived near the natural forest habitats of mosquitoes expressed no fear of mosquito bites, and to them, mosquitoes are harmless just like other rain forest insects. It is more of a nuisance than a potential health threat. This is similar to the connotation of a “peaceful co-existence” relationship between mosquitoes and the daily life of people in Villacicencio-Colombia, where the community was reported to perceive mosquitoes as part of their daily life and without health implication [26]. In our study, such belief was reflected in their laidback preventive practices such as merely closing windows to prevent mosquitoes from entering their homes or turning on a fan to ward off mosquitoes. In contrast, participants from urban areas were more likely to express concerns about mosquito menace, taking extra precautions to keep mosquitoes away such as using insecticides, mosquito repellents and window netting. They were also more likely to mention taking extra preventive measures against mosquito breeding in their surrounding environment. The differences in behavior and perception of these populations could have been shaped by the higher incidence of dengue in urban in contrast to rural areas. It can thus be suggested that future interventions should also focus on the identification of effective strategies to impart information on the potential presence of harmful species of mosquitoes among the rural community. This is especially important in the rapidly developing regions where there is a rapid encroachment of urbanization in the rural areas.

In our study, many expressed frustration on the lack of a concerted effort to prevent mosquito breeding by other residents in their community. Despite all efforts to prevent mosquitoes from breeding within their premises, many professed that they did not benefit from their preventive actions. This finding is in congruent with the construct of perceived benefit of the HBM. Participants' responses did not imply that such low perceived benefit impacts on their preventive measures; nevertheless, they may possibly provide socially desirable responses. The lack of concerted community efforts could potentially result in the lesser preventive engagements among those who actively practice preventive measures as they may not find meaningful results for the own efforts. The finding entails the importance of constant and permanent advocacy of social participation and concerted community action in dengue prevention and control, as reported in a previous study [27]. Findings from the study presented here, adds to the body of evidence supporting the importance of social mobilization and communication for sustainable dengue prevention and control [28], [29], [30].

The self-efficacy construct of the HBM mapped to emergent themes in many participants of diverse demographic backgrounds across all FGDs. Participants responded lack of self efficacy to consistently practicing preventive behaviours. Many admitted that in the event that there were no reports of dengue outbreak in their community, their efficacy in taking appropriate dengue preventive measures slackened.

In our study, we noted that media reports on an outbreak of dengue also impacted on the treatment-seeking behaviours of the study participants. As media coverage and increased rates of reporting of disease events were found to greatly influence public perception of risk [31], and in addition to the established relationship between perceived risk and actual practice of protective behaviours, constant public enlightenment related to the risk of dengue in the community, during both inter-epidemic and epidemic periods, would be beneficial. Further, educational intervention to increase perceptions of the threat of dengue is essential.

Our study results suggest that many were aware that one should seek prompt medical care when dengue is suspected. Nevertheless, most do not realize that even without taking any special remedy almost all DF spontaneously resolved after 5 days following onset of symptoms except in cases of DHF/DSS. This study, however, found that traditional medicine and home remedies play a central role in treatment-seeking. The unavailability of specific treatments for dengue also leads to reliance upon traditional folk remedies. The bases of trust in complementary medicine were perceived helpfulness via word of mouth messages or personal experience, and belief that the “natural” origins of traditional medicine and home remedies are harmless. Perception of efficacy of home remedies in relieving DF symptoms also shaped participants' perception of severity of dengue. Some ethnic groups have unique traditional practices and having an understanding of these is important for healthcare providers to encourage active communication with patients regarding the potential benefits and/or adverse effects of unconventional medical care. The perceived effectiveness of unconventional treatment cited by the participants warrants the need to enlighten the public to limit their reliance on unproven alternative treatments. Results also indicate that knowing about dengue infection occur in the community is an influential cue to action in spurring people to seek immediate medical attention if fever develops.

Lastly, it is important to note that while not specifically an HBM construct, knowledge is often incorporated in the HBM framework as knowledge about a disease or behavioral risk factors for a disease can positively promote individual's engagement behaviors to protect against the disease [5]. On the contrary, as indicated above, despite good knowledge about DF and prevention and control of dengue, practice of protective behaviors were more dependent on the beliefs based on the HBM. Therefore, this may imply that HBM should be use as the predominant theory driving intervention strategies to reduce dengue. Consequently, this is a research area that warrants further empirical investigation. Our ongoing quantitative national survey currently underway would be able to offer more conclusive evidence.

In interpreting these results, caution must be exercised when drawing conclusions beyond the sample assessed in the present study owing to the qualitative nature of the study [32]. Further investigation for generalisation of findings using quantitative methods is warranted. Nevertheless, the strength of this qualitative study lies in its large sample of focus group, furthermore the sample comprised of participants from a diverse demographic background, which closely resemble the general population in Klang Valley, Malaysia. Secondly, all information obtained from the discussion was self-reported, thus self-reporting bias towards socially desirable responses might exist. Other limitation includes the bias introduced by using only one coder. Lastly, although the results may imply health belief model's association with dengue preventive behaviors, the appropriateness of attitudes conceptualized by the HBM in the case of dengue, where repetitive preventive measures are to be performed on a daily basis and, in particular, the outbreak of dengue is seasonal, have raised concerns [33]. Despite these methodological caveats, the study presents an in-depth understanding of perceptions and their associated preventive behaviours which fills a gap in the relatively scarce literature on the influence of HBM on dengue in dengue endemic region.

### Conclusion

From the present study, several conclusions can be inferred. Firstly, with regard to knowledge about dengue, the study found that despite good knowledge about DF and prevention and control of dengue, preventive practices to control the vector were influenced by the main constructs of health belief in the HBM. The evidence from this study suggests that some basic information on the pathophysiology of DF and DHF/DSS may be beneficial. The study found that low perceived susceptibility forms part of the threat for young adults and the elderly. Reasons for the low perceived susceptibility to dengue infection emerged as two themes, namely perceived natural ability to withstand infection and low risk of coming into contact with Aedes mosquitoes. Self-efficacy has been found to be a critical barrier to self-regulatory prevention practices which could be enhanced with a consistent message about dengue. As perceived susceptibility and severity influences self-efficacy, education should be specific about high vulnerability to dengue and severity of DHF/DSS and the importance of avoiding secondary dengue infection.

The current findings add substantially to the understanding of health beliefs in prevention and control of dengue in dengue endemic region. Therefore, the HBM factors could be used as the theoretical basis of a health educational programme on dengue fever prevention and control in Malaysia. Unconventional treatment practices, including using home remedies and traditional medicine distinctive to cultural beliefs are prominent, thus warrant consideration when designing education messages of dengue for the region. In short, results obtained may be used to contextualize barriers to, or facilitators of, the adoption of dengue prevention behaviours in Malaysia.
